# Carboplatin with intravenous and subsequent oral administration of vinorelbine in resected non-small-cell-lung cancer in real-world set-up

**DOI:** 10.1371/journal.pone.0181803

**Published:** 2017-07-21

**Authors:** Vítězslav Kolek, Ivona Grygárková, Leona Koubková, Jana Skřičková, Jiřina Švecová, Dimka Sixtová, Jiří Bartoš, Aleš Tichopád

**Affiliations:** 1 Department of Respiratory Medicine, University Hospital, Olomouc, Czech Republic; 2 Department of Pneumology, University Hospital, Praha-Motol, Czech Republic; 3 Department of Respiratory Diseases and Tuberculosis, University Hospital Brno, Brno, Czech Republic; 4 Department of Oncology, Hospital Tabor, Tabor, Czech Republic; 5 Department of Respiratory Diseases, Memorial Thomayer Hospital, Praha, Czech Republic; 6 Department of Oncology, Regional Hospital Liberec, Liberec, Czech Republic; 7 Kantar Health s.r.o., Praha, Czech Republic; Universidade do Algarve Departamento de Ciencias Biomedicas e Medicina, PORTUGAL

## Abstract

**Objectives:**

Adjuvant cisplatin-based chemotherapy is recommended for routine use in patients with Stage IIA, IIB or IIIA non-small cell lung cancer (NSCLC) after complete resection. Results obtained for Stage IB were not conclusive. While vinorelbine plus cisplatin is the preferred choice after resection, combining vinorelbine with carboplatin promises improved compliance and delivery of drugs due to lower toxicity. We evaluated the impact of this option on treatment compliance and survival under real-world conditions.

**Material and methods:**

A prospective, single-arm, multicenter, non-interventional study evaluated the tolerability, dose intensity and survival resulting from adjuvant use of intravenous carboplatin (AUC 5 on day 1) with vinorelbine administered both intravenously (25 mg/m^2^ on day 1) and orally (60 mg/m^2^ on day 8) within four cycles of 21 days each. A total of 74 patients with a median age of 64 years were observed.

**Results:**

The mean number of accomplished cycles was 3.78, and 62 patients (83.7%) completed all four planned cycles. Relative dose intensity for carboplatin was 88.9%, for intravenous vinorelbine 93.1%, and for oral vinorelbine 83.2%. Median follow-up was 4.73 years. Median disease-specific survival (DSS) was 7.63 years, median overall survival (OS) was 5.90 years, median disease-free survival (DFS0) was 4.43 years, and five-year survival was 56.2%. TNM stage of disease significantly affected DSS and OS. Favorable survival was observed in females, nonsmokers, patients aged over 65 years, patient with prior lobectomy, patients with tumor of squamous histology, and those who finished the planned therapy, but the differences were non-significant.

**Conclusion:**

Adjuvant carboplatin with vinorelbine switched from intravenous to oral administration was shown to be a favorable regimen with regard to tolerability and safety. Compliance to therapy was high, and survival parameters were promising, showing that applied regimen can be another potential option for adjuvant chemotherapy in patients with NSCLC.

## Introduction

Lung carcinoma is the leading cause of cancer deaths. Radical surgery of non-small cell lung cancer (NSCLC) is an optimal solution with favorable survival outcomes. Nevertheless, at the time of surgery more than half of patients have already developed micrometastases in the blood or distant organs, resulting in significantly reduced survival. Approximately half of surgically treated patients with early-stage NSCLC die of this disease eventually [1]. Five-year survival after resection in patients with Stage I NSCLC is 50%, 46% in Stage IIA and 36% in Stage IIB. It drops to 24% in Stage IIIA [2,3]. The primary goal of adjuvant therapy in NSCLC is to eradicate micrometastases, reducing the rate of distant metastases and thus increasing disease-free survival (DFS) and overall survival (OS) following resection. Evidence of therapeutic benefit was available following the meta-analysis NSCLC-CG-MA [4]. Recently several large, randomized adjuvant trials conducted with cisplatin (CDDP) were performed [5–9], and key findings from these studies were pooled and analyzed in the Lung Adjuvant Cisplatin Evaluation (LACE) [10]. Over the 5.2-year follow-up, this study reported a hazard ratio of 0.89 for overall survival (OS) and 5.4% improvement in five-year OS for those on CDDP-based adjuvant therapy. Adjuvant CDDP-based chemotherapy is recommended for routine use in patients with Stage IIA, IIB and IIIA NSCLC after complete resection. Results obtained for Stage IB patients were not conclusive. As shown in the LACE meta-analysis, vinorelbine with CDDP is a preferable regimen in treating NSCLC after surgery [10]. The favorable results for use of CDDP in adjuvant therapy were further supported by two other meta-analyses with absolute five-year survival gains of 4% [11,12]. Some obviously unfavorable outcomes associated with the use of CDDP within the LACE dataset included treatment-related deaths, all planned cycles were administered in only 50% of patients, and the achieved relative dose intensity was rather low, all indicating that safety and tolerability were major unresolved issues associated with the CDDP-based regimens used [10]. Only one Phase III study was conducted with carboplatin (CBDCA) combined with paclitaxel in NSCLC patients in which no treatment-related deaths were reported and generally better safety and tolerability was observed [13]. Unlike other large adjuvant studies, this study only enrolled Stage IB patients. The effectiveness results, however, provide rather ambiguous answers. CBDCA could be considered a less toxic and hence more tolerable option and as such could be offered to patients with advanced NSCLC. CBDCA is preferred in elderly patients as well as in patients with comorbidities. Oral vinorelbine was shown to deliver better comfort than intravenous application and is hence preferred by patients [14]. Effectiveness and tolerability of oral vinorelbine with platinum in advanced NSCLC is comparable to intravenous application according to randomized studies [15,16]. It is advisable that the choice between CDDP or CBDCA in the adjuvant setting also involves dialogue between the physician and patient with respect to the patient’s preference, anticipated outcomes and risk of poor tolerability. With regard to the stage of disease and potential side effects, patient compliance should be critically considered as a key factor directly influencing outcomes. The evidence from a real-world setting involving patients’ options between CDDP and CBDCA combined with vinorelbine is missing.

## Methods

### Objectives

The objective of the study was to describe the outcomes of an adjuvant CBDCA-based chemotherapy in combination with vinorelbine administered first intravenously and then orally within a non-interventional real-world setting and facilitate comparison with historical cohorts published elsewhere. In this regard, dose delivery, toxicity and compliance to chemotherapy were evaluated as primary variables, along with dose intensity, DFS and OS.

### Design and patient cohort

This was a prospective, multicenter, non-interventional study for evaluation of the adjuvant use of carboplatin and vinorelbine in management of NSCLC patients in routine clinical practice. Consecutive chemotherapy-naive 18- to 75-year-old patients after complete resection of Stage IB, IIA, IIB and IIIA NSCLC (6th TNM) suitable for chemotherapy (ECOG 0–1, le >3.5 G/l, neu >1.5 G/l, tr >100 G/l, AST <1.5 x N, ALT <1.5 x N) who, given the choice, opted to be treated with CBDCA and initial intravenous followed by subsequent oral vinorelbine were followed up from January 12, 2005, until September 5, 2008. In accordance with common recommendations, the treatment was not given to those with expected poor compliance, marked neurological impairment, impaired hematogenesis, diseases preventing thoracotomy, or another malignancy. Throughout the study, intravenous CBDCA (AUC = 5 on Day 1) with vinorelbine was administered both intravenously (25 mg/m^2^ on Day 1) and orally (60 mg/m^2^ on Day 8). Four cycles of the 21-day regimen were planned. In parallel, physicians had the option to use a CDDP-based treatment. Growth factors (GMCSF) were applied, when neutropenia dropped under <1.0 cells x 10^9^ /L. In case of absolute neutrophil count ≤ 1·5 cells x 10⁹/L or platelet count ≤ 75 cells x 10⁹/L chemotherapy was postponed for one week, in case of continuing toxicity the cycle was canceled. In case of repeated neutropenia and/or thrombocytopenia dose reduction was applied (vinorelbine 60 mg p.o./m^2^ on day D1,D8 without reescalation, carboplatin 50% of dose). In case of neurotoxicity Gr 2, 50% vinorelbine reduction was applied, when neurotoxicity of Gr 3 appeared, vinorelbine was stopped.

### Ethical and legal frame

The study was initiated by the investigator–the Department of Respiratory Medicine, Faculty of Medicine and Dentistry, Palacky University Olomouc and University Hospital Olomouc, Czech Republic, and was conducted as non-interventional. Evaluation and approval of the protocol by an ethical committee was part of the research fund approval process coordinated by the Grant Agency of the Czech Republic. This study was reviewed and approved by an Institutional Review Board of the University Hospital in Olomouc/Faculty of Medicine, Palacky University in Czech Republic and participants provided written informed consent.

### Baseline examination

NSCLC was confirmed histologically prior to or shortly after surgery. Age and gender were recorded along with data of first diagnosis of NSCLC. The baseline stage of disease was established using chest X-ray, CT and bronchoscopy. Distant metastases were determined using CT of adrenals and upper abdomen, liver scintigraphy or liver sonography. Bone scintigraphy was conducted where increased alkaline phosphatases or calcinemia was observed or pain in bones was reported by the patient. Where neurological impairments were present, brain CT was undertaken. The staging as obtained was critical for surgery indication and classification of the patient. The extent of the surgery was considered histologically. Surgery greater than or of the same size as lobectomy was considered radical. The time interval between diagnosis and surgery could not be greater than one month followed by chemotherapy not later than six weeks after surgery. Only those with radical resection were further followed up to provide data.

### Medication

Within two to six weeks following the surgery, patients received treatment with CBDCA, comorbidities and/or patient’s own acceptance. CBDCA with a target AUC = 5 was administered together with intravenous vinorelbine 25 mg/m^2^ on Day 1 followed by oral vinorelbine 60mg/m^2^ on Day 8 (switch of administration). In total, four 21-day cycles were used. Dose of CBDCA was calculated by Calvert’s formula using a calculated creatinine clearance with the Cockcroft–Gault formula. Premedication was undertaken using dexamethasone or setrons when needed. Treatment was applied on out-treatment basis.

### Follow-up

The stage of disease was further reassessed at the end of the fourth cycle of chemotherapy using chest X-ray and CT of chest and upper abdomen. Bronchoscopy was performed only where endobronchial progression was suspected. Bone scintigraphy was conducted where hypercalcinemia or increased alkaline phosphatases were observed or bone pain was reported by patient. Brain CT was performed if neurological impairments were present. A detailed restaging was then further performed in half-year intervals and where recurrent disease was suspected. Generally, patients requiring radiotherapy after surgery were not enrolled for this study.

### Data analysis

The study was an observational, non-interventional study intended for evaluation of the adjuvant use of carboplatin and vinorelbine in management of NSCLC patients in routine clinical practice. A formal sample size calculation was not performed. The estimated number of participants was based on the expected number of potentially suitable NSCLC patients, indicated to the evaluated chemotherapy, at all participating sites within the pre-defined observation period. The data analysis used SAS for Windows (SAS Institute Inc., Cary, NC, USA), and graphs were created with SW Statistica (StatSoft, Inc., Tulsa, OK, USA). All data as collected in the CRF were described using descriptive statistics; in particular, categorical data were described using absolute and relative frequencies, and metric data were described by arithmetic mean, standard deviation, minimum, maximum and median. A survival analysis for OS, DSS and DFS were computed using the Kaplan-Meier method. All three survival types were further tested using a log-rank test to compare the survival distributions in the presence of the following factors and covariates: age, gender, histology, stage, smoking, type of surgery, early termination of medication, percentage intensity vinorelbine IV, percentage intensity vinorelbine PO, and percentage intensity CBDCA. Received dose intensity (RDI) was calculated for both drugs used with respect to mode of administration along with the median follow-up duration [17].

## Results

### Cohort

A total of 74 patients were enrolled in the study, 53 (72%) males and 21 (28%) females. The median age was 64 years, ranging from 48 to 77 years. Most patients were smokers (41 patients, 55.41%), 27 patients were ex-smokers (36.49%), and six were non-smokers (8.11%).

### Histology and tumor stages

Tumor was squamous in 46 cases (62.16%), adenocarcinoma in 22 (29.73%), giant cell in four (5.41%), and not otherwise specified in two (2.70%) patients. Pneumonectomy was performed in 26 patients (35.14%), lobectomy in 43 (58.11%) and bilobectomy in five patients (6.76%). With regard to the observed histopathological tumor stages, 19 patients were Stage IB (25.68%), eight in Stage IIA (10.81%), 22 in Stage IIB in 22 (29.73%) and 25 in Stage IIIA (33.78%). Various pathological tumor-node-metastasis (pTNM) stages were observed as follows: T1N1M0 in eight (10.81%), T1N2M0 in three (4.05%), T2N0M0 in 19 (25.68%), T2N1M0 in 18 (24.32%), T2N2M0 in 14 (18.92%), T3N0M0 in three (4.05%), T3N1M0 in six (8.11%), and T3N2M0 in three (4.05%) patients.

### Safety and tolerability

Out of 74 patients, 62 (83.78%) accomplished all four planned cycles of CBDCA chemotherapy with vinorelbine. Four patients accomplished only two (5.41%) and eight patients only three (10.81%) cycles. The mean number of delivered cycles was 3.78. The most frequently observed hematological Grade 3/4 toxicities were neutropenia in 19 patients (25.68%), leukopenia in 12 (16.22%), anemia in six (8.11%) and trombocytopenia in two (2.70%), and febrile neutropenia in two (2.70%). Non-hematological toxicities observed were alopecia in nine patients (12.16%), nausea in three (4.05%), diarrhea in three (4.05%), and nephrotoxicity in one (1.35%). Growth-stimulating factor was applied in seven patients (5.18%) and erythropoietin in three patients (2.22%). No treatment-related deaths were reported. RDI achieved for CBDCA was 88.9% (95% CI: 85.68%-92.04%), RDI for intravenous vinorelbine was 93.1% (95% CI: 90.49%-95.75%), and RDI for oral vinorelbine was 83.2% (95% CI: 79.83%-86.66%).

### Survival

**Fig 1:**
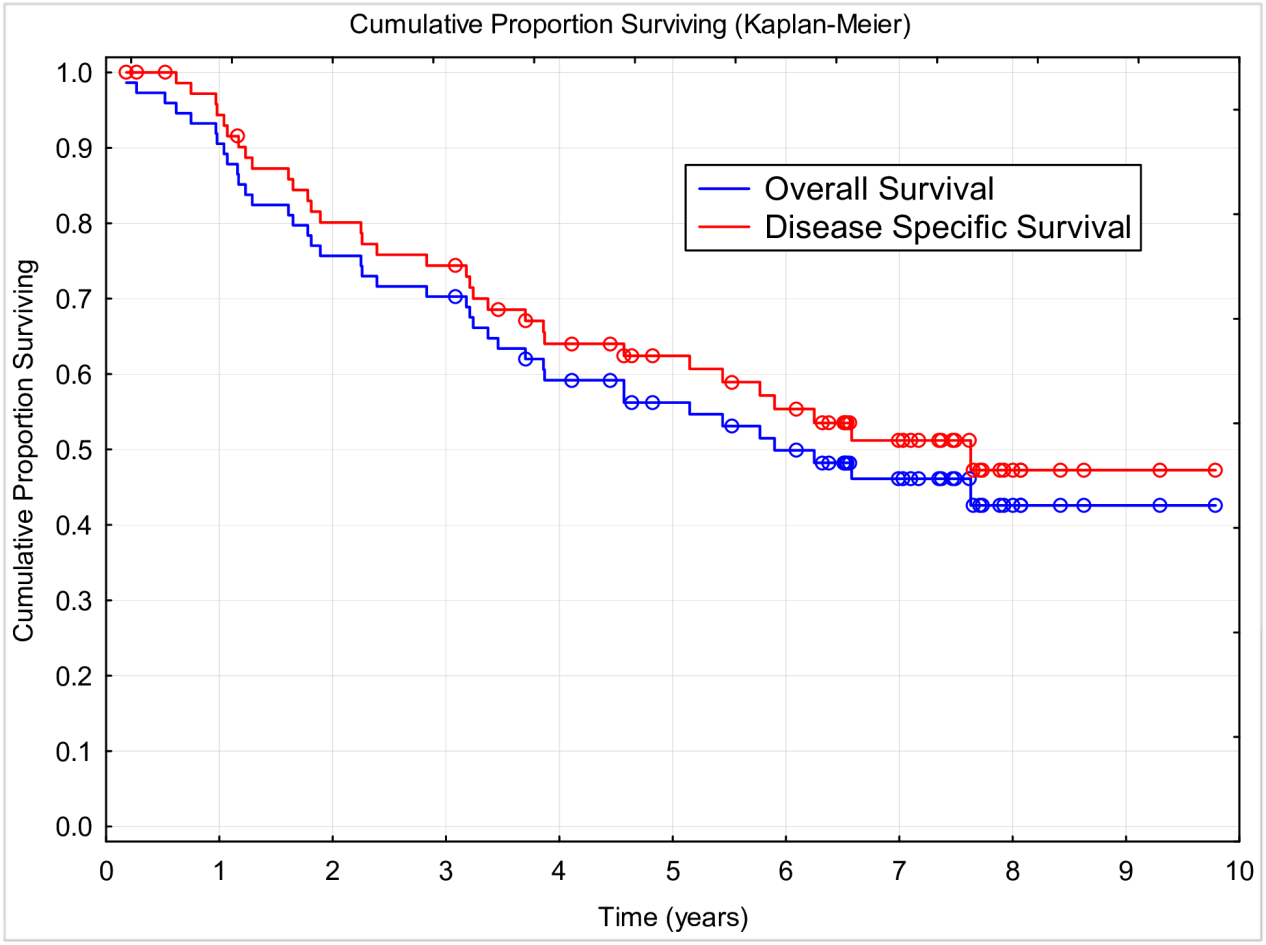
Overall survival and disease specific survival.

**Fig 2:**
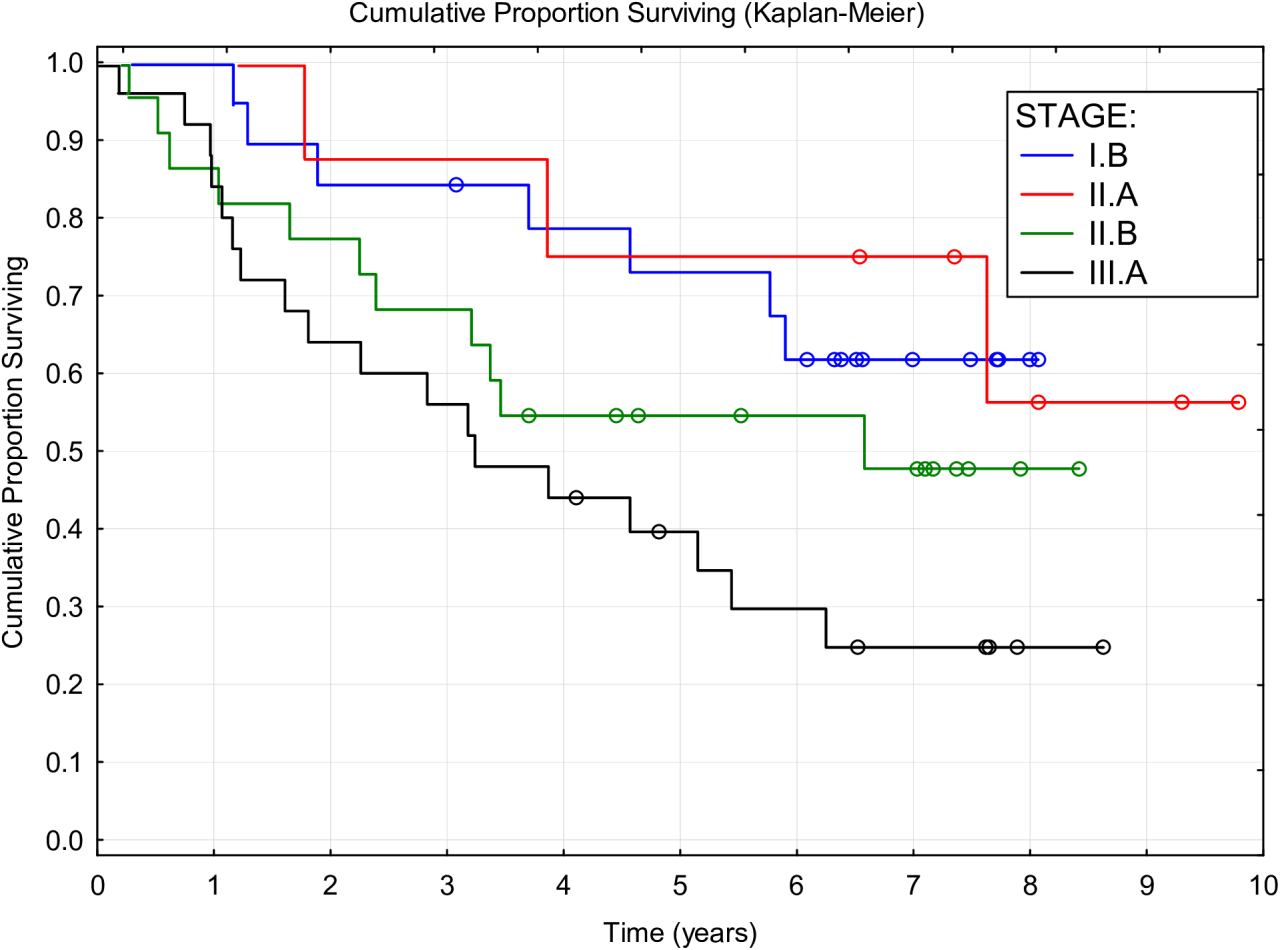
Overall survival by disease stage.

**Fig 3:**
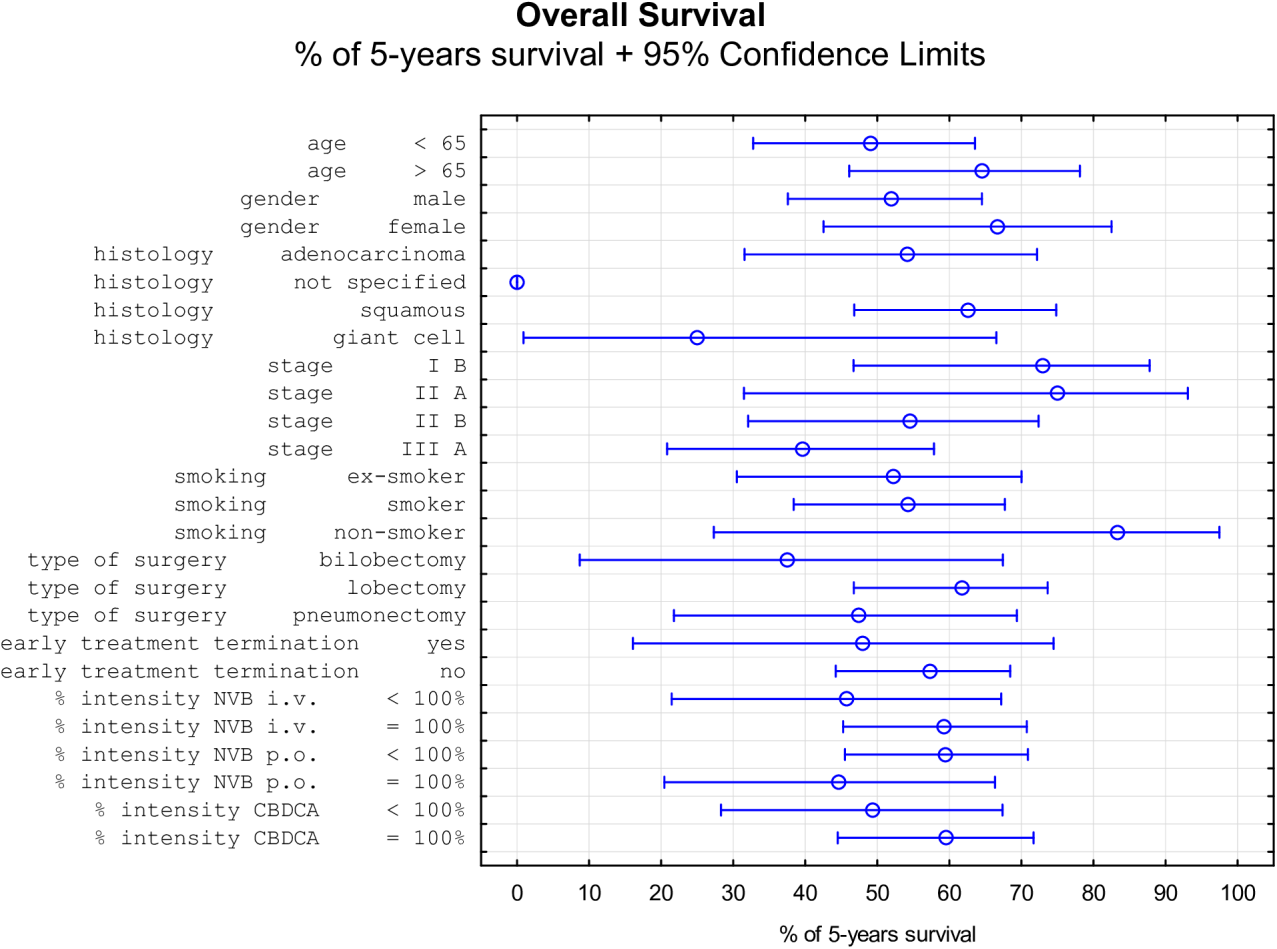
Tree plot showing percentage of 5-year overall survival with 95% confidence limits. Data presented in Fig 3 are derived from Table 1.

During the follow-up period of a median of 4.73 years (2 months to 9.79 years), 30 (40.54%) patients died of NSCLC, six (8.11%) died of other causes, and three (4.05%) were lost to follow-up; and 35 (47.3%) patients were still alive at the end of the study. Median DSS was 7.63 years (95% CI: 4.57 to NR), median OS was 5.9 years (95% CI, 3.7 to NR), and median DFS was 4.43 years (95% CI: 2.53 to NR). Three-year survival of 70.3% and five-year survival of 56.2% were attained. The Kaplan-Meier curves of OS and DSS are shown in Fig 1; the five-year DFS was 48.42%. The effects of all factors and covariates are shown in Tables 1–3. The stage of disease was shown to significantly affect the OS and DSS but not the DFS. Other than this, none of the factors or covariates detected had any significant effect on any of the three survival outcomes. The effect of disease stage on OS is shown in Fig 2. Non-significant differences in survival were observed in females, nonsmokers, patients of age over 65 years, after lobectomy, with tumor of squamous histology, and those who finished the planned therapy (Fig 3).

**Table 1:** Overall survival stratified by selected factors and covariates.

Factor	Cut off	N (total)	N (events)	5-years survival	Log Rank Test
**age**	< 65	40	21	0.491	0.601
	> 65	34	18	0.646	-
**gender**	male	53	30	0.520	0.242
	female	21	9	0.667	-
**histology**	adenocarcinoma	22	13	0.542	0.136
	not specified	2	2	0.000	-
	squamous	46	21	0.626	-
	giant cell	4	3	0.250	-
**stage**	I B	19	7	0.730	0.042
	II A	8	3	0.750	-
	II B	22	11	0.546	-
	III A	25	18	0.396	-
**smoking**	ex-smoker	23	16	0.522	0.181
	smoker	45	21	0.543	-
	non-smoker	6	2	0.833	-
**type of surgery**	bilobectomy	8	6	0.375	0.275
	lobectomy	50	25	0.617	-
	pneumonectomy	16	8	0.474	-
**early treatment termination**	yes	10	5	0.480	0.916
	no	64	34	0.574	-
**% intensity NVB i.v.**	< 100%	29	12	0.458	0.789
	= 100%	45	30	0.592	-
**% intensity NVB p.o.**	< 100%	61	39	0.594	0.861
	= 100%	13	8	0.446	-
**% intensity CBDCA.**	< 100%	42	29	0.494	0.720
	= 100%	32	14	0.595	-

**Table 2:** Disease specific survival stratified by selected factors and covariates.

Factor	Cut off	N (total)	N (events)	5-years survival	Log Rank Test
**age**	< 65	40	19	0.517	0.350
	> 65	34	14	0.751	-
**gender**	male	53	24	0.606	0.564
	female	21	21	0.667	-
**histology**	adenocarcinoma	22	12	0.591	0.498
	not specified	2	1	-	-
	squamous	46	18	0.670	-
	giant cell	4	2	0.333	-
**stage**	I B	19	6	0.786	0.043
	II A	8	3	0.750	-
	II B	22	8	0.650	-
	III A	25	16	0.434	-
**smoking**	ex-smoker	23	14	0.576	0.196
	smoker	45	17	0.616	-
	non-smoker	6	2	0.833	-
**type of surgery**	bilobectomy	8	6	0.375	0.103
	lobectomy	50	21	0.678	-
	pneumonectomy	16	6	0.578	-
**early treatment termination**	yes	10	5	0.480	0.771
	no	64	28	0.647	-
**% intensity NVB i.v.**	< 100%	29	12	0.521	0.637
	= 100%	45	30	0.651	-
**% intensity NVB p.o.**	< 100%	61	39	0.658	0.835
	= 100%	13	8	0.510	-
**% intensity CBDCA.**	< 100%	42	29	0.629	0.852
	= 100%	32	14	0.621	-

**Table 3:** Disease free survival stratified by selected factors and covariates.

Factor	Cut off	N (total)	N (events)	5-years survival	Log Rank Test
**age**	< 65	40	20	0.469	0.392
	> 65	34	14	0.653	-
**gender**	male	53	23	0.564	0.800
	female	21	11	0.524	-
**histology**	adenocarcinoma	22	12	0.496	0.589
	not specified	2	1	-	-
	squamous	46	19	0.599	-
	giant cell	4	2	0.333	-
**stage**	I B	19	7	0.684	0.352
	II A	8	4	0.625	-
	II B	22	9	0.538	-
	III A	25	14	0.415	-
**smoking**	ex-smoker	23	12	0.466	0.579
	smoker	45	19	0.578	-
	non-smoker	6	3	0.667	-
**type of surgery**	bilobectomy	8	6	0.375	0.128
	lobectomy	50	21	0.593	-
	pneumonectomy	16	7	0.506	-
**early treatment termination**	yes	10	5	0.467	0.746
	no	64	29	0.567	-
**% intensity NVB i.v.**	< 100%	29	12	0.584	0.644
	= 100%	45	30	0.544	-
**% intensity NVB p.o.**	< 100%	61	39	0.598	0.253
	= 100%	13	8	0.412	-
**% intensity CBDCA.**	< 100%	42	29	0.624	0.809
	= 100%	32	14	0.521	-

## Discussion

Because several large, aggregated studies repeatedly support the use of platinum-based compound as an effective strategy in eradication of cancer cells, they have recently become the therapeutic basis of adjuvant treatment of NSCLC [10–12]. The limited tolerability is alarming; i.e., 33% of patients received two or fewer cycles of therapy [10]. Among the main causes for discontinuation were toxic reactions, rejection of treatment by the patient, or even death caused by chemotherapy [10]. The achieved relative dose intensity ranged from 23% to 50%. A minimum CDDP dose of 240 mg/m^2^ was used in only 59% of patients; hence, it is obvious that approximately half of all patients received lower dose than planned. The three largest recent trials, IALT, ANITA, and JBR.10 reported treatment-related deaths ranging from 0.8% to 1.7% [7,8,18]. In patients, older than 80 years it was 3.1% [19]. This raises a severe ethical concern in light of the fact that the patients who died from chemotherapy were largely cured–with rather favorable survival prognosis. In published meta-analyses comparing CBDCA and CDDP in inoperable stages of NSCLC, slightly better objective outcomes with CDDP were reported. Superior survival was reported for new chemotherapeutic regimens in non-squamous lung cancer [20,21]. So far no studies have compared CBDCA and CDDP in adjuvant therapy in the early stages of NSCLC [22]. The data on tolerance collected through our real-world use study are similar to those reported in CALGB9633, a single CBDCA-based adjuvant Phase III study with patients treated with CBDCA (AUC = 6) and paclitaxel (200 mg/m^2^) [13]. It included 344 patients with Stage IB disease and despite the fact that it was considered as negative, it demonstrated a significant survival difference in favor of adjuvant chemotherapy for patients who had tumors ≥ 4 cm in diameter (HR = 0.69; p = 0.043). This result was in accordance with findings of the large meta-analyses on CDDP chemotherapy [10,13]. Furthermore, in the CALGB9633 study, no patient died from chemotherapy, and 86% of patients completed all four cycles of chemotherapy, although the dose had to be reduced in 34% of those patients. Several smaller studies with CBDCA and paclitaxel, vinorelbine or docetaxel have been published with comparable results [23,24]. The dose intensity of docetaxel and CBDCA was 87.5% and 86%, respectively, and the full dose was achieved in 74% of patients within 12 weeks [24]. Observational studies on CBDCA showed benefits in older patients with organ dysfunction, neuropathy or other comorbidities. There were also fewer reports of infections, dehydration and vomiting compared with the use of CDDP [18,25]. CBDCA is an alternative to CDDP in patients with cardiac disorders, renal insufficiency or hearing loss. Furthermore, a shorter infusion period with no need for pre- or posthydration renders carboplatin more suitable for outpatient-based regimens. This can be one of the reasons for preference of CBDCA in real-world studies performed in Canada and United States [26,27]. Only one retrospective study exists in which oral vinorelbine 60 mg/m^2^ (Day 1 and 8) was used with CDDP 75 mg/m^2^ every three weeks in the adjuvant setting [28]. Non-statistical differences comparing parenteral administration of vinorelbine were found. The combination of switched parenteral to oral vinorelbine with CBDCA showed good efficacy and tolerability in advanced NSCLC in a Phase II study, and safety profiles for early disease in the present study on early NSCLC were comparable [29]. The present multicenter prospective study can help answer whether the use of CBDCA at a higher dose intensity could deliver a similar effect than CDDP-based regimens with a lower dose intensity. The good tolerance in the adjuvant setting could be well supported by the 84% of patients completing all four cycles, which is much higher than the numbers reported elsewhere for CDDP-based protocols [7,8]. Obtained survival parameters are encouraging, but limitations of the present study are obviously great. The small number of patients and the lack of direct comparison with other therapeutic regimens bring the demand of further randomized studies to establish further evidence concerning outcome. There are many open questions regarding the optimal approach in adjuvant chemotherapy of NSCLC, and finding better tolerated regimens is one of them. This research remains prospective because large studies using biological adjuvant therapy have not yet yielded positive results [30,31]. There are also discussions considering whether different adjuvant trials without good biomarkers could bring more robust evidence for complete response and survival prolongation or whether the focus should be toward neoadjuvant (or perioperative chemotherapy) trials to hasten drug development, achieve objective responses and increase resectability [32,33]. Selective, more effective and more tolerable adjuvant therapy is the challenge for future research.

## Conclusion

Adjuvant chemotherapy with carboplatin and vinorelbine given intravenously on Day 1 and orally on Day 8 every three weeks appeared to be a convenient and tolerable therapy in radically resected NSCLC. This prospective study provided high dose intensity of delivered drugs and facilitated acceptable survival with high treatment completion.
